# Influence of Extraction Techniques on Almond Oil Quality: A Comparative Study of Solvent-Extracted and Commercial Products

**DOI:** 10.3390/molecules30173519

**Published:** 2025-08-28

**Authors:** Mariola Kozłowska, Diana Mańko-Jurkowska, Bartłomiej Zieniuk, Magdalena Rudzińska

**Affiliations:** 1 Department of Chemistry, Institute of Food Sciences, Warsaw University of Life Sciences-SGGW, 159C Nowoursynowska Str., 02-776 Warsaw, Poland; mariola_kozlowska@sggw.edu.pl (M.K.); bartlomiej_zieniuk@sggw.edu.pl (B.Z.); 2 Department of Food Technology of Plant Origin, Poznań University of Life Sciences, 28 Wojska Polskiego Str., 60-637 Poznań, Poland

**Keywords:** almond oil, fatty acid profile, health indices, oil quality, oxidative stability

## Abstract

The aim of the study was to compare the quality of almond oils obtained using different extraction methods, including cold solvent extraction, Soxhlet extraction, and the Folch method. Oils were extracted from four commercially available almond-based products—unpeeled almonds, blanched almonds, almond flakes, and almond protein concentrate—and compared with a commercially refined almond oil. The extracted oils were analyzed for their fatty acid (FA) composition and selected quality parameters, including acid value, peroxide value, *p*-anisidine value, the TOTOX index, and specific extinction coefficients (K_232_ and K_268_). Based on the FA profiles, health-related indices such as atherogenic index, thrombogenic index, and hypocholesterolemic/hypercholesterolemic ratio were also calculated. Additionally, the oxidative stability of the oils was assessed using an accelerated method—pressure differential scanning calorimetry. The obtained results demonstrated that the extraction method had a stronger influence on almond oil quality than the type of raw material. Oil extracted from unpeeled almonds using Soxhlet and cold solvent techniques showed better oxidative stability and more favorable FA profiles, while oils obtained using the Folch method and commercial refined oils exhibited higher levels of primary and secondary oxidation products. These findings were further supported by statistical analyses, which revealed distinct groupings based on oxidation indices and lipid composition.

## 1. Introduction

The almond tree, also known as the almond (*Prunus dulcis*), is a fruit tree belonging to the Rosaceae family. It is native to the Middle East and Central Asia, but is now cultivated in regions with a Mediterranean climate, as well as in California, Iran, and Spain [1]. The almond tree is mainly cultivated for its edible seeds called almonds. Almonds have an elongated, ellipsoidal shape, and their surface is smooth, light beige, surrounded by brown skin. There are two varieties: sweet almonds (*P. dulcis* var. *dulcis*) and bitter almonds (*P. dulcis* var. *amara*) [2]. Both varieties, along with raspberries, apples, and pears, belong to the Rosaceae family, but sweet almonds are most often intended for consumption raw or roasted as a snack or addition to dishes. The market offers almonds with skin, blanched almonds, almond flakes, and products based on almonds, including flour, butter, almond milk, oil, and protein preparations [3]. In the food industry, they are used in confectionery and bakery products, for the production of beverages, pastes, and salads, and also as an addition to dietary snacks and high-protein products [4]. Bitter almonds, on the other hand, are not intended for direct consumption due to the presence of amygdalin, which is degraded to glucose, benzaldehyde, responsible for their bitter flavor, and toxic hydrogen cyanide [5]. The kernels are the edible part of the almonds and are considered a good source of minerals such as K, P, Ca, and Mg, which play a key role in the proper functioning of muscles, preventing them from cramps that can occur after intense physical exercise. In addition, almond seeds contain vitamin E, known for its antioxidant properties, and B vitamins, which are responsible for the proper functioning of the nervous system [6]. Almonds are also a good source of proteins (~20%), carbohydrates (~20%), dietary fiber (12.5%), and fat (~50%) [7]. Due to their high nutritional content, almonds are often used as a raw material for oil extraction, as almond oil (AO) is considered a valuable ingredient in both food and cosmetic industries due to its composition and health-promoting properties.

Literature reports indicate that the method of oil extraction from plant materials affects various properties of the oil, including chemical composition, nutritional value, purity, quality, oxidative stability, and shelf life [8]. The process of extracting oil from almonds is similar to that of extracting oils from other nuts or seeds. It can be done by different methods, depending on the desired quality of the oil and the scale of its production [9]. One of the methods used is cold-pressing, which is considered an ecological method that does not result in a qualitative change in the oil obtained [10]. In turn, conducting this process at a higher temperature can contribute to forming sulfur compounds that reduce its quality. To prevent this and improve purity, the oil should be refined. AO can also be obtained using various extraction methods, among which solvent extraction provides the highest industrial efficiency. The most commonly used solvent is hexane, one of the relatively cheap and readily available solvents that allows selective extraction of fat without affecting the presence of other components, such as fiber. However, it is necessary to remove its traces before consuming the oil so that it meets food safety standards [8].

AO has a rich and diverse chemical composition. It contains sterols, tocopherols, squalene, a significant content of monounsaturated fatty acids (MUFA), the primary representative of which is oleic acid (C18:1), a moderate content of linoleic acid (C18:2), and a relatively low level of saturated fatty acids (SFA) [11,12], which may be associated with a reduced risk of cardiovascular disease, as well as potential benefits in other diseases such as hypertension and diabetes [9,13]. Due to its mild, slightly nutty flavor, AO can be used for dressings, as an addition to salads, and in the production of almond butter or nut pastes as an agent that imparts smoothness and facilitates the right consistency, making the mass more homogeneous and easier to spread. Moreover, due to its favorable lipid profile, characterized by a high share of mono- and polyunsaturated FAs, it can act as a functional substitute for traditional fats in the technology of confectionery and bakery products, contributing to the improvement of their nutritional value.

AOs’ yield, physicochemical, and bioactive properties may vary depending on the isolation technique used. Özcan et al. [14] compared them using cold pressing and Soxhlet extraction for three almond varieties. In turn, Sayah et al. [15] determined them for oils obtained from almonds with and without skin using three extraction methods (supercritical CO_2_, cold press, and solvent extraction). An important factor influencing the quality of the AO obtained is also the type, degree of ripeness, and the form in which the almonds were used to get it, i.e., whether they were used in the form of raw almonds or whether they had previously been roasted or blanched. Roasting and blanching are pre-treatment methods that can significantly affect the raw material’s physicochemical properties and nutritional value, which in consequence may affect the chemical composition, quality, and functional properties of the resulting oil [16].

In this study, three solvent-based extraction techniques (Folch, Soxhlet, and cold solvent extraction) were applied to obtain oils from four different commercially available almond products, including unpeeled almonds, blanched almonds, almond flakes, and almond protein concentrate (APC). The nutritional properties, quality parameters, and oxidative stability of the extracted oils were determined and compared to those of commercially available refined AO.

## 2. Results and Discussion

### 2.1. Oil Extraction Yield

Literature data indicate that the oil extraction yield from seeds depends on several factors, including the type and composition of the raw material, the extraction method and conditions, as well as the nature and polarity of the solvent used. Moreover, the physical characteristics of the raw material, such as particle size, moisture content, and the presence of seed coat, can significantly affect oil accessibility during extraction [11,17,18].

In the present study, AO yield varied depending on both the type of raw material used (unpeeled almonds, blanched almonds, almond flakes, and APC) and the extraction method applied (Soxhlet, Folch, and cold solvent extraction), as shown in Table 1.

Soxhlet extraction proved to be the most efficient method for obtaining oil from blanched almonds, yielding nearly 86% of the oil content declared by the manufacturer. In contrast, the Soxhlet method was the least effective for APC: almost twice as much oil was obtained from APC using the Folch method (~7.51%) compared to Soxhlet extraction (~3.78%). Interestingly, the fat content extracted from APC using the Folch method (approximately 7.51%) exceeded the value declared by the manufacturer (6.6%). This discrepancy may be attributed to differences in analytical methodology, as the Folch method enables the extraction of both free and bound lipids, potentially capturing a broader range of lipid fractions than standard procedures used for nutritional labelling [19,20]. This is likely related to the higher polarity of the chloroform:methanol mixture, which facilitates the recovery of lipids bound to proteins and membranes—components that are particularly abundant in protein-rich materials such as APC.

Literature data indicate that the polarity of solvents used for extraction may play an important role in lipid recovery efficiency. The non-polar nature of hexane, used in both Soxhlet extraction and cold solvent extraction, favors the recovery of neutral lipids (triacylglycerols), which constitute the major fraction of AO [19]. Literature reports confirm that Soxhlet extraction can be more efficient than the Folch method, for example, in the case of walnut oil extraction [21]. In contrast, the more polar biphasic system of chloroform and methanol in the Folch method also enables the recovery of polar lipids (e.g., phospholipids, glycolipids). In addition, the cold maceration principle applied in the Folch method may reduce matrix degradation and facilitate the extraction of biologically active compounds, including antioxidants, due to the nature of the solvents [19,20,21]. Each of the extraction methods—Soxhlet, Folch, and cold solvent extraction—can influence oil yield and quality in positive or negative ways. While they may enhance lipid recovery and extract a wider range of compounds, they may also result in the co-extraction of undesirable substances or alter oil composition due to prolonged exposure to heat or solvents [21]. In the present study, differences in oil yield among the three extraction methods reflected not only variations in solvent polarity and extraction conditions, but also suggested the influence of other factors inherent to the raw material. This complexity underlines that solvent polarity alone cannot fully explain the observed differences in oil yield.

For unpeeled almonds, the highest oil yield was obtained by cold *n*-hexane extraction (~43.43%), whereas this method resulted in the lowest yield for blanched almonds (~37.02%). The opposite trend was observed for Soxhlet extraction, with the highest yield recorded for blanched almonds (~46.42%) and the lowest for unpeeled ones (~37.56%). This may be explained by the thermal degradation of heat-sensitive components present in almond skin, which could reduce oil recovery during Soxhlet extraction.

Other authors have reported different trends for oil extraction from unpeeled almonds. Miraliakbari and Shahidi [22] observed lower yields with cold *n*-hexane extraction (51.2%) compared to a chloroform/methanol mixture (53.5%). Similarly, Krzyczkowska et al. [20] reported higher oil yield with the Folch method (50.24%) than with Soxhlet extraction (43.75%). These discrepancies in extraction yields among studies may result from differences in raw material characteristics (such as variety or origin), solvent-to-solids ratios, and specific extraction conditions, including contact time. Both literature data and the results obtained in this study highlight the need to consider oil extraction as a process influenced by numerous interacting variables, rather than a single standardized procedure.

### 2.2. Fatty Acid Profile and Health Indices of Almond Oils

Plant oils are primarily composed of triacylglycerols; therefore, the FA composition and distribution within triacylglycerol molecules represent a key characteristic of the lipid fraction in plant seeds [23]. Table 2 shows the FA profiles of the tested AOs extracted by different methods and compared with the commercial, refined AO. The results revealed that oleic acid was the predominant FA in all analyzed oils, with its lowest content observed in the commercial AO (60.92%) and the highest in the oil obtained from blanched almonds via Folch extraction (69.59%). Oleic acid is the most common MUFA in commonly consumed foods. It enhances the activity of low-density lipoprotein (LDL) receptors and lowers serum cholesterol level [24]. As such, it plays a crucial role in the human diet by regulating cholesterol metabolism and contributing to a reduced risk of cardiovascular diseases. According to literature data, oils derived from olives, avocados, macadamia nuts, and hazelnuts also exhibit high oleic acid content, often exceeding 50% of their total FA composition [25,26].

The second most abundant FA in AO was linoleic acid, with the highest share found in the commercial AO (27.60%) and the lowest in the oil extracted from APC using the Folch method (19.50%). Although linoleic acid is the most abundant PUFA in the human diet, it is classified as essential, as it cannot be synthesized by mammals. Linoleic acid is vital for maintaining cell membrane integrity, regulating inflammatory processes, and supporting proper skin function and overall physiological homeostasis [27]. Similar to AO, other oils such as olive oil, avocado oil, and high-oleic sunflower oil also contain between 20% and 30% linoleic acid in their total FA profiles.

The FA composition of the analyzed AOs was compared with that reported by Maestri et al. [28]. The authors noted that AO typically contains oleic acid (50–80%), linoleic acid (10–26%), and palmitic acid (5–9%). The results obtained in this study fall within those reported ranges. The FA profile of all tested AOs was characterized by a high MUFA content (Table 2), with the highest share found in the oil extracted from blanched almonds using the cold solvent method (70.43%) and the lowest in commercial AO (61.35%). These values fall within the broad range (43.3–83%) reported by Ouzir et al. [8] for oils varying in almond origin and extraction method, as well as within the range reported by Rabadán et al. [29] for oils obtained from Spanish almond varieties (66–73.5%).

These findings are consistent with broader comparative studies evaluating MUFA levels across various plant oils. According to Tian et al. [30], AO exhibits the highest MUFA content (77.07%) among 28 tested plant oils, followed by olive oil (76.62%) and papaya seed oil (76.10%). Although MUFAs are less prone to oxidation than PUFAs, PUFAs are essential FAs that must be obtained through the diet. Both MUFAs and PUFAs play an important role in cardiovascular disease prevention.

Several dietary indices, such as the index of atherogenicity (AI), the thrombogenicity index (TI), and the hypocholesterolemic/hypercholesterolemic ratio (h/H), can be calculated based on FA profiles to assess the potential impact of dietary fats on cardiovascular health. It is recommended to consume products with a low AI (<1.0) and TI (<0.5), and a high h/H, as such profiles are associated with reduced total and LDL cholesterol levels in blood plasma. Conversely, consumption of products with a higher TI and a lower h/H ratio may increase the risk of cardiovascular diseases [23].

To provide a more comprehensive characterization of the analyzed oils, nutritional indices commonly used to assess potential health-promoting properties were calculated and are presented in Table S1 (Supplementary File). The tested AOs exhibited favorable nutritional and health-related properties, as reflected by very low AI values (0.07 and 0.08), low TI values (0.21 and 0.22, and 0.24 only for the Folch-extracted oil from APC), and a high h/H ratio (11.98–13.36). The most favorable profiles, characterized by very low AI and TI values and a high h/H ratio, were observed for the AOs obtained from APC using the Soxhlet method and the cold solvent extraction method. The obtained results suggest that, based on the applied indices, these oils may be considered the most beneficial from a cardiovascular health perspective.

Khalili Tilami and Kouřimská [31] compared AI and TI values across nine different categories of fats and oils, including nut oils. The values obtained in the present study were consistent with those reported for AO by the authors (AI = 0.07 and TI = 0.21). Notably, the AI values observed for AOs were among the lowest across all evaluated oils and comparable to those of hemp oil, chia seed oil, and hazelnut oil—products associated with low cardiovascular risk and recognized health benefits.

The TI values reported here fell within the range typical for traditional nut oils (0.16–0.35) and were similar to those for soybean oil (0.21) [31], blackcurrant seed oil, and hemp oil (0.23) [32].

The h/H index reflects the potential impact of dietary FAs on cardiovascular health by representing the balance between hypocholesterolemic FAs (*cis*-C18:1 and PUFA) and hypercholesterolemic FAs (C12:0, C14:0, and C16:0) [33]. The lowest h/H value was observed for the Folch-extracted oil from APC (11.98), whereas the highest h/H values were found in oil also obtained from APC but extracted using the Soxhlet method (13.36) and the cold solvent extraction method (13.32). The h/H value for refined, commercial oil was 12.64. The high h/H ratios calculated for the tested AOs indicate pronounced health-promoting potential, comparable to those of rosehip oil (11.81) [33], camelina oil (11.7–14.7) [34], dill seed oil (12.56), and blackcurrant seed oil (13.82) [32].

Given their promising nutritional profile, the overall quality of the AOs was further evaluated based on key physicochemical parameters, as discussed in the next section.

### 2.3. Determination of Quality Parameters

Quality parameters are among the most important factors to assess in oils, as they determine their resistance to degradation, storage stability, and functional properties. One of the main contributors to oil deterioration is the oxidation of TAGs, which occurs upon exposure to light or ambient oxygen. This process leads to the breakdown of TAG molecules and the release of free FAs [21].

The acid value (AV) serves as an indicator of oil freshness and the extent of hydrolysis, reflecting the concentration of free FAs present. According to Codex Alimentarius standards, the AV of cold-pressed vegetable oils should not exceed 4 mg KOH per gram of oil, while for refined oils, the limit is 0.6 mg KOH/g oil [35]. In the current study, almost all tested oils complied with these standards (Table 3), except for the oil extracted from APC using the Folch method, which showed a significantly elevated AV (16.77 mg KOH/g oil). When comparing the influence of extraction method on AV, the highest values were consistently observed for oils extracted using the Folch method, regardless of the raw material. In contrast, oils obtained using the cold-solvent and Soxhlet methods yielded comparable, and notably lower, AVs. In terms of raw material, the lowest AV was found in commercial refined oil (0.24 mg KOH/g oil), which is consistent with the known effect of refining processes that remove impurities and reduce rancidity-promoting compounds [36,37]. Interestingly, similar low AVs were obtained in oils extracted from unpeeled almonds and APC using both the cold-solvent and Soxhlet methods.

On the other hand, higher AVs were observed in oils derived from blanched almonds (0.89–1.50 mg KOH/g oil), and even higher in oils from almond flakes (1.37–2.04 mg KOH/g oil). These findings suggest that blanched and processed almond products are more susceptible to unfavorable environmental factors such as heat and humidity, which promote hydrolytic degradation. Moreover, the structural disruption occurring during blanching and flaking enhances the exposure of oils to oxygen and lipolytic enzymes, thereby accelerating hydrolysis and oxidation [38]. Additionally, the increased surface area in flaked almonds facilitates greater contact between the oil and ambient air and moisture, further contributing to AV elevation.

Following the evaluation of AV, the peroxide value (PV) provides further insight into the early stages of lipid oxidation. PV reflects the extent of oxidative changes in oil, and it is directly proportional to the concentration of primary oil oxidation products, namely peroxides. According to the Codex Alimentarius, the maximum permissible PV is 15 mEq O_2_/kg for cold-pressed vegetable oils and 10 mEq O_2_/kg for refined vegetable oils [39]. Almost all tested oils met these standards (Table 3), with the exception of oil extracted using the Folch method from unpeeled almonds (10.19 mEq O_2_/kg) and APC (13.07 mEq O_2_/kg).

Similarly to AV, PV was also consistently highest in oils extracted by the Folch method, regardless of the raw material. However, for PV, more favourable (lower) values were observed in oils extracted using the Soxhlet method (1.77–2.25 mEq O_2_/kg) compared to those obtained via the cold solvent method (2.05–3.87 mEq O_2_/kg). In contrast, an opposite trend was noted for the *p*-anisidine value (*p*-AnV), which reflects the presence of secondary oxidation products such as aldehydes. In this case, lower *p*-AnV was observed in oils extracted by the cold solvent method (0.30–0.75) than by Soxhlet extraction (0.51–1.54) (Table 3). These findings can be attributed to the thermal conditions of Soxhlet extraction, which is conducted at the boiling point of the solvent. Under such conditions, primary oxidation products (peroxides) may decompose into secondary products [40], leading to a reduction in PV and a corresponding increase in *p*-AnV. As with AV and PV, the highest *p*-AnVs were recorded for oils extracted using the Folch method. Interestingly, when the raw material underwent pre-treatment processes such as blanching or flaking, the oil extracted from it via the Folch method showed a PV nearly half that of the oil from unpeeled almonds, but *p*-AnV nearly twice as high. Additionally, the total oxidation index (TOTOX), which accounts for both primary and secondary oxidation products and thus provides a comprehensive measure of oxidative deterioration, was also highest in oils obtained by the Folch method (ranging from 13.92 to 30.11) (Table 3).

Similarly, refined oil showed relatively high values of quality parameters: PV (9.65 mEq O_2_/kg), *p*-AnV (3.24), and TOTOX (22.53). Although refining, which includes several steps (neutralization, bleaching, degumming, and deodorization), typically reduces the AVs and PVs by removing free FAs and primary oxidation products [36], the *p*-AnV may either decrease or, under certain conditions (e.g., excessively high temperature or prolonged deodorization), increase due to the formation or incomplete removal of secondary oxidation compounds [41,42]. Simultaneously elevated PV and *p*-AnV in the tested commercial oil may indicate that it had undergone both primary and secondary oxidation, reflecting advanced lipid degradation and reduced oxidative stability. This could result from factors such as improper storage, prolonged storage time, low natural antioxidant content, secondary contamination, or the presence of oxidation catalysts [43].

El Bernoussi et al. [5] reported that cold-pressed sweet AO degraded significantly during storage at 60 °C for four 4 weeks, with the PV increasing from 2.4 to 24.6 mEq O_2_/kg. Similalry, Sidhu et al. [44] observed marked increases in PV (from 2.66, 2.55 and 2.95 to 9.02, 8.06, and 9.88 mEq O_2_/kg), *p*-AnV (from 3.67, 3.71, and 3.63 to 16.88, 16.43, 15.89), and TOTOX (from 8.99, 8.81, and 9.53 to 27.93, 26.67, and 27.99) in AOs from Australian, American, and Iranian origins, respectively, after 21 days of storage at elevated temperature. In contrast, self-extracted AOs from almond flour tested by Dias et al. [10] showed favorable quality parameters (PV = 1.8–2.0 mEq/kg oil, *p*-AnV = 0.1–0.4, and TOTOX = 4.0–4.3) compared to those reported in the present study. It is worth noting that the authors used environmentally friendly extraction and recovery methods. Similarly low PVs, ranging from 1.9 to 3.2 mEq/kg, were reported for different varieties of sweet AO self-obtained by Melhaoui et al. [12]. These findings suggest that the good quality parameters observed in the self-extracted oils may be attributed to their freshness and the fact that they were tested immediately after extraction. The commercial oil was analyzed during its labeled shelf life. However, the conditions of its transport and storage prior to the study were unknown and may have adversely affected its quality.

Other parameters used in the oil quality assessment are specific extinction coefficients denoted as K_232_ and K_268_. While the K_232_ coefficient is an indicator of the presence of conjugated dienes, which are formed as a result of the primary oxidation of unsaturated FAs, especially linoleic acid, the K_268_ coefficient is used to assess the content of conjugated trienes and secondary oxidation products, such as aldehydes and ketones [45]. Increased K_232_ values are the first signal of deteriorating oil quality, and K_268_ indicates the presence of more persistent and undesirable degradation compounds. The K_232_ values presented in Table 3 showed that for all the extracted oils, they were in the range of 1.89 ± 0.06–4.97 ± 0.19. Regardless of the type of extraction method used, the lowest K_232_ values were observed for oils from unpeeled almonds, amounting to 2.00 ± 0.01 (cold solvent extraction), 1.89 ± 0.06 (Soxhlet method), and 2.87 ± 0.01 (Folch method), respectively. It seems that this may be influenced by the presence of protective compounds in the brown skin of the almond [15]. In turn, oils extracted from APC were characterized by the highest K_232_ values, which were 3.42 ± 0.07 (cold solvent extraction), 3.18 ± 0.04 (Soxhlet method), and 4.97 ± 0.19 (Folch method), respectively. The K_232_ values obtained for these types of oils corresponded to a similar trend noted when discussing the PV, which may indicate the beginning of the oxidation process of the linoleic acid contained in them. Considering the method of oil extraction from the raw material used, it was observed that slightly lower K_232_ values were determined for oils obtained by the Soxhlet method, and higher ones by the Folch method. In contrast, no statistically significant differences were observed in the K_232_ values obtained for oils from unpeeled almonds and almond flakes extracted by the Soxhlet and cold solvent methods, in which the primary solvent was *n*-hexane. However, slight differences were observed when blanched almonds and APC were used as raw materials. The K_232_ values obtained for the oils extracted from almonds with *n*-hexane using both the Soxhlet apparatus and the cold solvent extraction method were similar to those obtained for the oils extracted from almonds with petroleum ether as extractant and not subjected to electron beam treatment [46].

About the K_268_ coefficient, the values obtained for the studied oils were low and close to each other. They ranged from 0.10 ± 0.01 for oil obtained from almond flakes by the cold solvent extraction method to 0.86 ± 0.02 for oil from APC obtained by the Folch method. No statistically significant differences were observed in the K_268_ values for the studied oils obtained using the same raw material, the Soxhlet method, and cold solvent extraction. These differences were significant when comparing oils obtained from the same material but using the Folch method. There, an increase in the K_268_ extinction coefficient was found. In general, the values of the two extinction coefficients K_232_ and K_268_ were higher when the oils were extracted by the Folch method, i.e., using a solvent mixture that could be too aggressive, leading to co-extraction of compounds susceptible to oxidation. What is worth emphasizing is that the highest values of K_232_ and K_268_, as well as the previously discussed PV, *p*-AnV, and TOTOX parameters, were observed for the commercial oil studied. This may indicate lipid degradation and reduced oxidative stability in this sample.

Overall, the results indicate that differences in quality parameters among the extraction methods were not solely attributable to solvent polarity. As mentioned earlier, both cold solvent and Soxhlet extraction employed *n*-hexane as the solvent, whereas the Folch method used a chloroform: methanol mixture, which differs substantially in polarity. This variation in solvent polarity likely influenced the extraction efficiency of peroxides and other oxidation products. While the extraction of highly polar oxidation products is unlikely to be the main cause of yield differences, our results indicate that such compounds may still be recovered and contribute to the higher quality parameters observed in Folch-extracted oils. In addition, factors such as extraction temperature and raw material characteristics, including prior processing (e.g., blanching or flaking), also played a significant role. These findings highlight the multifactorial nature of oil quality determination and the need to interpret oxidation indices in the context of both solvent properties and process-related variables, suggesting that both the type of raw material and the extraction method may also influence the oxidative stability of AO, which is examined in the following section.

### 2.4. Oxidative Stability by Pressure Differential Scanning Calorimetry

Lipid oxidation plays a crucial role in determining the final quality and nutritional value of food products, as it is the primary reaction responsible for their degradation. Oxidative stability is therefore a key quality indicator for edible oils [47]. In food chemistry, pressure differential scanning calorimetry (PDSC) is an accelerated calorimetric technique used to assess the oxidative stability of plant oils [34]. The oxidation time determined by PDSC (τ_max_) corresponds to the maximum oxidative changes.

**Figure 1:**
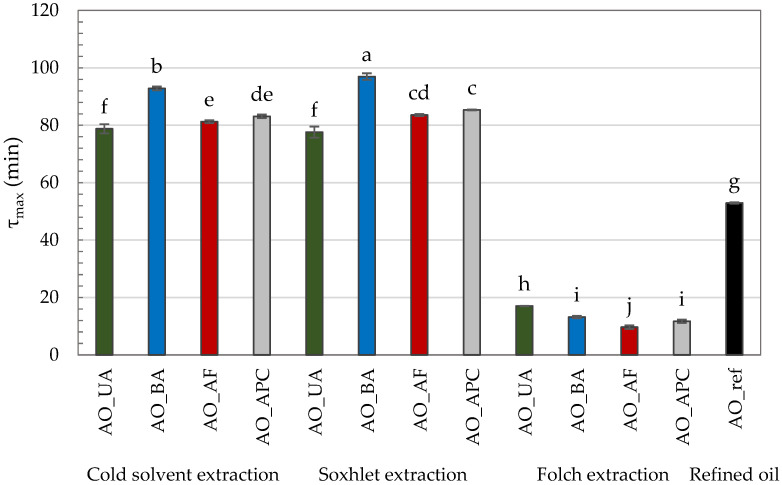
PDSC oxidation time (τ_max_) of commercial and self-extracted almond oils, where: AO_UA—oil extracted from unpeeled almonds; AO_BA—oil extracted from blanched almonds; AO_AF—oil extracted from almond flakes; AO_APC—oil extracted from almond protein concentrate; AO_ref—commercial, refined oil. Lowercase letters (a–j) indicate a significant difference at the significance level of 0.05 (Tukey HSD).

The τ_max_ values for the tested AOs varied considerably depending on the extraction method and the raw material variant (Figure 1). The shortest τ_max_ values (9.67–17.06 min) were recorded for oils extracted using the Folch method, indicating their markedly lower oxidation resistance. This observation is in line with their higher PV, *p*-AnV, and TOTOX values, which reflect both a lower initial resistance to oxidation and a more advanced oxidative deterioration, further accelerated under PDSC conditions.

In contrast, τ_max_ was significantly higher and comparable for oils extracted by cold solvent and Soxhlet methods, with Soxhlet-extracted oils consistently showing the highest τ_max_ values. The extended τ_max_ in Soxhlet-extracted oils may be attributed to high extraction temperatures, which can inactivate prooxidant enzymes such as lipoxygenase [38,48]. Under cold extraction, such enzymes may remain active and initiate oxidative processes during oil preparation. Moreover, elevated temperatures during Soxhlet extraction may enhance the release of lipophilic antioxidants from the plant matrix [49]. Qi et al. [11] demonstrated that both phytosterols and tocopherol with tocotrienols have a significant correlation with oxidation induction time (*p* < 0.01).

An exception to this trend was observed for oils derived from unpeeled almonds, where no statistically significant differences in τ_max_ were found between extraction methods. However, even among hexane-extracted oils, this variant exhibited the lowest τ_max_ (~78 min), suggesting the least oxidative stability. Conversely, the highest τ_max_ values were observed for oils obtained from blanched almonds—92.82 min (cold solvent extraction) and 96.96 min (Soxhlet extraction)—indicating the greatest oxidative resistance. Interestingly, this cannot be directly linked to better conventional quality indicators, as these oils did not display superior PV, *p*-AnV, and TOTOX values. This discrepancy highlights a key distinction: while PV, *p*-AnV, and TOTOX reflect the current oxidative state of the oil, τ_max_ measures its resistance to future oxidation under accelerated conditions. Thus, an oil may already be partially oxidized yet still retain compounds that slow further oxidation. The enhanced oxidative stability of oils from blanched almonds may be explained by the blanching process itself, which may remove prooxidant components present in the peel and thermally inactivate oxidative enzymes [38,50]. Although there is a remarkable amount of antioxidants concentrated in the peels of nuts, they can interact with each other and exhibit either synergistic or inhibitory effects [51].

The value of τ_max_ is closely related to the FA composition of the oil. It is well-established that the presence of multiple bonds significantly accelerates the rate of oil oxidation; for example, linoleic acid oxidizes 10–40 times faster than oleic acid, while α-linolenic acid oxidizes 2–4 times faster than linoleic acid [52]. In the tested AOs, α-linolenic acid was absent, and the percentage of oleic acid (60.92–69.79%) was significantly higher than that of linoleic acid (19.50–27.60%). Notably, the highest proportion of linoleic acid was found in the commercial oil (27.60%), which, along with elevated quality parameters, likely contributed to its markedly lower τ_max_ value (52.91 min) compared to the hexane-extracted oils.

Lipid oxidation is a complex phenomenon influenced by many factors, including processing methods and parameters, exposure to light, oxygen, and elevated temperatures during storage, as well as the presence of non-glyceride components with pro- or antioxidant activity, and the overall quality of the raw material used [23]. Among these factors, FA composition and the method used to obtain the oil play a particularly important role in determining oxidative stability.

Presented results clearly indicate that τ_max_ is shaped by a multifactorial interplay between intrinsic oil characteristics, with particular emphasis on the FA profile, and the method used to obtain it. These findings are consistent with literature data obtained under identical PDSC conditions, which allows a direct and reliable comparison of τ_max_ values between different oils. Ratusz et al. [53] reported a τ_max_ of 56.73 min for refined rapeseed oil and 24.55 min for sunflower oil, consistent with their respective FA profiles: rapeseed oil (~60% oleic acid, 20% linoleic acid) and sunflower oil (~30% oleic acid, ~60% linoleic acid) [54]. Similarly, Siol et al. [23] found that refined watermelon seed oil had a markedly lower τ_max_ (23.88 min) than oil extracted with hexane at room temperature (76.55 min), highlighting the strong influence of the oil obtaining method on oxidative stability.

In the context of these findings, AO—particularly when obtained from blanched kernels—exhibits relatively high oxidative stability compared with other edible oils, positioning it favourably within the broader spectrum of vegetable oils analysed under PDSC conditions. Moreover, the application of PDSC provides an additional and valuable tool for benchmarking oxidative stability across different oils, offering predictive insights that complement conventional quality indices such as PV, *p*-AnV, and TOTOX.

### 2.5. Multivariate Analysis of Almond Oil Quality Parameters

Multivariate statistical analyses were conducted to complement the experimental assessment of AO quality. These methods aimed to uncover patterns and relationships among samples based on their composition and quality parameters.

**Figure 2:**
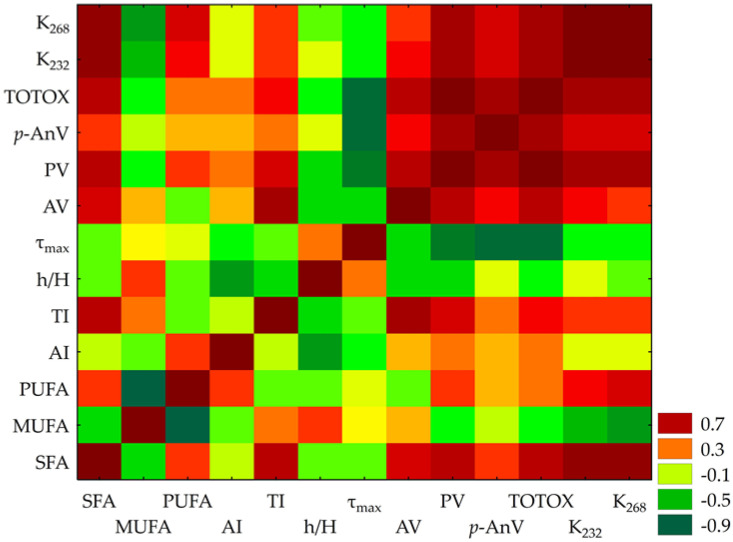
Heatmap of Pearson correlation coefficients among lipid stability indices and fatty acid profiles (n = 13), where: SFA—saturated fatty acids, MUFA—monounsaturated fatty acids; PUFA—polyunsaturated fatty acids; AI—index of atherogenicity; TI—index of thrombogenicity; h/H—hypocholesterolaemic/hypercholesteraemic index; AV—acid value; PV—peroxide value; *p*-AnV—*p*-anisidine value; TOTOX—total oxidation index; K_232_ and K_268_—the specific extinction coefficients; τ_max_—PDSC oxidation time. Red indicates positive correlations while green denotes negative correlations, with significance at *p* < 0.05.

The correlation matrix (Figure 2) revealed significant relationships among 13 variables. Notably, PV and TOTOX exhibited an exceptionally strong positive correlation (r = 0.993), suggesting near-perfect alignment in their measurement of oxidative degradation. Similarly, K_232_ and K_268_, both absorbance indices linked to conjugated dienes and trienes, showed a strong positive association (r = 0.933). PV demonstrated robust correlations with K_232_ (r = 0.751) and K_268_ (r = 0.757), reinforcing their role as markers of oxidative processes.

A striking inverse relationship emerged between *p*-AnV and τ_max_ (r = −0.893), suggesting that shorter τ_max_ corresponds with higher secondary oxidation products.

SFAs demonstrated moderate-to-strong positive correlations with K_232_ (r = 0.859) and K_268_ (r = 0.866). Conversely, MUFA showed a weak negative correlation with the atherogenic index (r = −0.283). Meanwhile, AI and h/H exhibited a moderate negative correlation (r = −0.600), indicating a significant relationship between FA profiles and cardiovascular risk indices.

**Figure 3:**
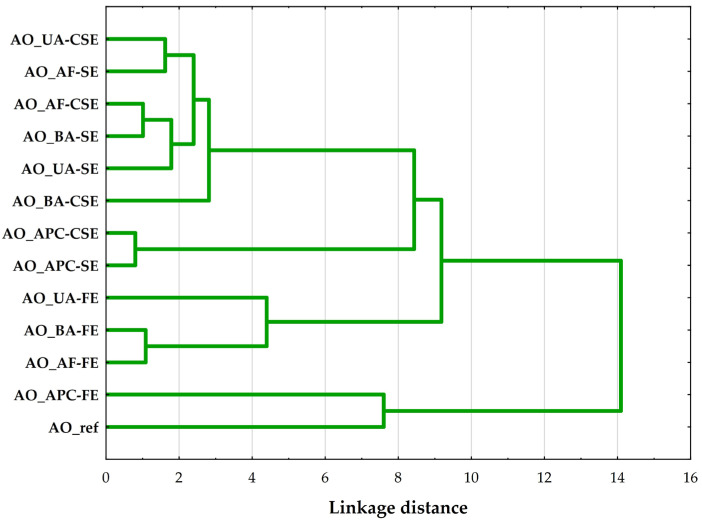
Hierarchical cluster analysis (Ward’s method, Euclidean distance) dendrogram of compared almond oils, where: AO_UA—oil extracted from unpeeled almonds; AO_BA—oil extracted from blanched almonds; AO_AF—oil extracted from almond flakes; AO_APC—oil extracted from almond protein concentrate; AO_ref—commercial, refined oil; CSE—cold solvent extraction; SE—Soxhlet extraction; FE—Folch extraction.

A hierarchical cluster analysis (HCA) was performed on the AO samples using Ward’s method with Euclidean distance as the similarity metric. The resulting dendrogram (Figure 3) shows the presence of four main clusters, suggesting significant compositional and quality differences among the AO samples.

The first cluster groups oils extracted using cold solvent and Soxhlet methods from whole almond forms (unpeeled, flaked, and blanched). These oils likely share similar FA profiles and low oxidative degradation, suggesting that such extraction conditions preserve the intrinsic properties of the source material. The similarity across different almond types indicates that the extraction technique has a greater influence on the oil’s profile than the almond’s processing form in this group [15].

The second cluster includes oils derived from APC, extracted using both cold solvent and Soxhlet methods. The close grouping reflects a unique compositional profile distinct from that of whole almond forms, likely due to prior protein isolation, which alters the matrix and residual lipid content. These oils exhibited a lower PUFA content compared to oils from whole almonds.

The oils in the third cluster were all obtained through Folch extraction, regardless of the form of the almond. The clustering indicates that the Folch method, which utilizes a chloroform–methanol solvent system, results in a distinct oil quality, possibly due to its higher extraction efficiency for polar lipids [22]. These differences suggest that the extraction method plays a significant role in shaping the oil characteristics in this case, overshadowing variations attributed to almond form.

The final and most distinct cluster includes Folch-extracted oil from APC and a commercial refined AO. Despite their different origins and extraction processes, these oils clustered together due to their significantly altered composition. Notably, this group was characterized by low overall oil quality, as indicated by elevated PV, *p*-AnV, and TOTOX index, along with K_232_ and K_268_ indices showing higher levels of primary and secondary oxidation products.

**Figure 4:**
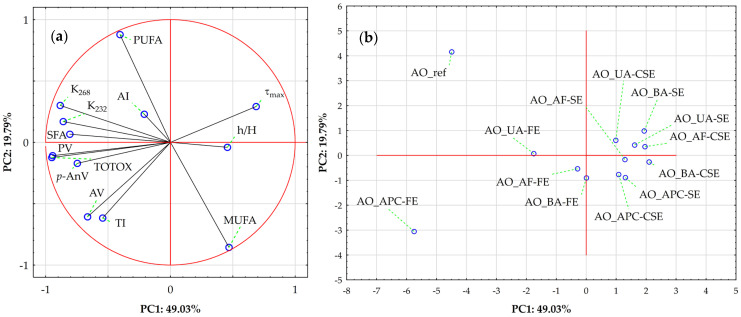
Principal component analysis (PCA) of almond oils based on lipid stability indices and fatty acid profiles. (**a**) Loading plot for PC1 vs. PC2; where symbol designations are as in Figure 2. (**b**) Score plot showing sample distribution on PC1 vs. PC2; where symbol designations are as in Figure 3.

Finally, Figure 4 illustrates the results of a Principal Component Analysis (PCA) to explore sample variation driven by lipid stability indices and FA profiles. Together, the first two principal components explain 68.82% of the total variance (PC1: 49.03%; PC2: 19.79%). The correlation circle plot (Figure 4a) shows PC1 is mainly influenced by strong negative loadings from TOTOX (−0.951), PV (−0.945), K_268_ (−0.884), K_232_ (−0.860), SFA (−0.807), *p*-AnV (−0.747), and AV (−0.664), and strong positive loadings from τ_max_ (0.687), MUFA (0.469), and h/H (0.457). This plot reveals distinct groupings of variables, with the length of vectors indicating each variable’s relative influence. A tight cluster of oxidation markers (TOTOX, PV, *p*-AnV, K_232_, K_268_, and SFA) mainly influences PC1 in the negative direction, emphasizing their strong positive correlation and role as key factors in variation linked to oxidative degradation. These markers are primary indicators of oil quality deterioration. In contrast, MUFA, τ_max_, and h/H are projected in the positive PC1 direction, directly opposing the oxidation markers, suggesting an inverse relationship with oxidation severity. PC2 is driven primarily by a strong positive loading from PUFA (0.878), with smaller positive contributions from τ_max_ (0.292) and K_268_ (0.301), and strong negative contributions from MUFA (−0.856), TI (−0.616), and AV (−0.607), indicating that PUFA variation occurs largely independently of the oxidation markers.

Then, the Score Plot (Figure 4b) visualizes sample distribution in the PC1-PC2 space. The extraction method (SE—Soxhlet extraction, FE—Folch extraction, CSE—cold solvent extraction) emerges as a key differentiator, with clear clustering visible despite some overlap—a finding strongly aligned with the hierarchical results (Figure 3). Refined and Folch extracted-protein concentrate oils occupy the extreme negative end of PC1, confirming their association with advanced oxidation. In contrast, samples near the center exhibit intermediate profiles.

## 3. Materials and Methods

### 3.1. Materials and Chemicals

Commercial, refined sweet AO (*Prunus dulcis*) of pharmaceutical quality (Greenaction, Kielce, Poland) was purchased within its shelf life and tested prior to expiration. The product originated from the USA.

Certified organic almonds, almond flakes, and blanched almonds were purchased from Bio Planet (Leszno, Poland). The unpeeled Italian almonds contained 22% protein, 53% fat (including 5.1% SFA), 3.9% carbohydrates (including 3.5% sugars), 13% fiber, and 0.03% salt. The blanched almonds (also from Italy) had a composition of 22% protein, 54% fat (including 3.9% SFA), and 5.4% carbohydrates (including 4.8% sugars). Spanish almond flakes contained 22% protein, 53% fat (5.1% SFA), 3.9% carbohydrates (3.5% sugars), and 13% fiber.

A commercially available APC (Smart Organic, Sofia, Bulgaria), with a minimum protein content of 50% (dry basis), was used in this study. According to the manufacturer’s data, APC contained 6.6% fat (0.6% SFA), 9.6% carbohydrates (9.0% sugars), 14% fiber, and 0.03% salt, and was obtained through a gentle, low-temperature, solvent-free extraction process.

All solvents and reagents used were of chromatographic or analytical grade, sourced from Avantor (Gliwice, Poland), except for standard compounds, which were supplied by Sigma–Aldrich (Saint Louis, MO, USA). All analyses were carried out after oil extraction and prior to the expiration date of commercial oil.

### 3.2. Methods

#### 3.2.1. Oil Extraction

Extractions were performed immediately after opening commercial products. Raw materials (unpeeled almonds, blanched almonds, and almond flakes) were ground separately using a laboratory mill to facilitate solvent penetration. Each extraction was carried out using 30 g of ground raw material or 60 g of protein powder.

#### Cold Solvent Method

Samples were extracted with 300 mL of *n*-hexane (200 mL for protein powder) by mechanical shaking at room temperature for 120 min. After extraction, the mixtures were centrifuged at 5000 rpm for 20 min. The supernatants were dried with anhydrous magnesium sulfate, which was then filtered off. Solvent was removed under reduced pressure at 40 °C using a rotary vacuum evaporator (Rotavapor^®^ R-300, BUCHI, Uster, Switzerland), followed by nitrogen purging to eliminate residual solvent.

#### Soxhlet Method

Samples were wrapped in filter paper, placed into extraction thimbles, and extracted with *n*-hexane (as in Section Cold Solvent Method) using a Soxhlet apparatus for 4 h at the solvent’s boiling point. After extraction, the solvent was removed by rotary evaporation under reduced pressure, and residual hexane was eliminated by purging with nitrogen.

#### Folch Method

Oil extraction was performed using the Folch method [55], modified according to Boselli and Caboni [56]. Samples were mixed with 100 mL of a chloroform:methanol (1:1, *v*/*v*) solution, shaken for several minutes, and incubated in a laboratory dryer at 60 °C for 20 min. After cooling, 100 mL of chloroform was added to re-extract remaining lipids, followed by mechanical shaking. The mixtures were filtered using a Büchner funnel to recover solid residues. Subsequently, 70 mL of aqueous potassium chloride was added, and the mixtures were left overnight at 4 °C. The following day, the biphasic systems were allowed to reach room temperature and were separated using a separatory funnel. The lower (chloroform) phase was collected and dried over anhydrous sodium sulfate for 2 h, then filtered to remove the drying agent. The solvent was evaporated under reduced pressure, and residual solvent was removed by purging the oil samples under a stream of nitrogen.

#### 3.2.2. Oil Yield Determination and Fatty Acid Composition Analysis

Oil yield from the raw materials was determined gravimetrically and expressed as a percentage, calculated as the ratio of the extracted oil mass to the initial sample mass, according to Equation (1):

(1) $$Oil\;yield(\%)=\frac{m_{o}}{m_{s}}\times 100$$

where *m_o_* is the mass of the extracted oil and *m_s_* is the mass of the raw material.

The FA composition of each oil sample was determined by gas chromatography using a YL6100 GC Clarity system (Young Lin Bldg., Anyang, Hogye-dong, Republic of Korea) equipped with a flame ionization detector and a BPX-70 capillary column (SGE Analytical Science, Milton Keynes, UK). FA methyl esters were prepared according to EN ISO 5509:2001 [57], and nitrogen was used as the carrier gas.

The chromatographic conditions were as follows: initial oven temperature of 70 °C (held for 30 s), increased to 160 °C at 15 °C/min, then to 200 °C at 1.1 °C/min, and finally to 225 °C at 30 °C/min (held for 1 min). The injector and detector temperatures were set at 225 °C and 250 °C, respectively.

FAs were identified by comparing their retention times with those of a standard FA methyl esters mixture (Supelco 37 Component FAME Mix, Sigma-Aldrich, Bellefonte, PA, USA). The relative content of each FA was expressed as a percentage of the total identified FAs.

#### 3.2.3. Health Indices of Oils

The FA composition was used to calculate the health-related lipid indices of the tested oils. The atherogenic index and thrombogenic index were calculated according to the equations proposed by Ulbricht and Southgate (Equations (2) and (3)) [58], while the hypocholesterolaemic/hypercholesterolaemic ratio was determined from Equation (4) [59]:

(2) $$AI=\;\frac{C12:0+\left(4\times C14:0\right)+C16:0}{\sum MUFA+\sum PUFA\;n-6+\sum PUFA\;n-3}$$

(3) $$TI=\frac{C14:0+C16:0+C18:0}{0.5\times \sum MUFA+\left(0.5\times \sum PUFA\;n-6\right)+\left(3\times \sum PUFA\;n-3\right)+\frac{\sum PUFA\;n-3}{\sum PUFA\;n-6}}$$

(4) $$h/H=\frac{cis-C18:1+\sum PUFA}{C12:0+C14:0+C16:0}$$

where: FA—fatty acids; UFA—unsaturated fatty acids; SFA—saturated fatty acids; MUFA—monounsaturated fatty acids; PUFA—polyunsaturated fatty acids. The calculations were based on the percentage area of each FA relative to the total FA content.

#### 3.2.4. Quality Parameters Determination

The physicochemical quality of the tested oils was evaluated based on standard lipid quality parameters. The AV, reflecting the extent of hydrolytic degradation, was determined using the AOCS Official Method Te 1a-64 [60]. The PV, representing the content of primary oxidation products, was assessed according to AOCS Cd 8b-90 [61]. Both AV and PV were measured using an automatic titrator (TitraLab AT1000 Series, Hach Lange, Wroclaw, Poland).

The degree of secondary oxidation was determined based on the *p*-anisidine value, following AOCS Cd 18–90 [62]. The TOTOX index was calculated using the following formula: TOTOX index = (2 × PV) + *p*-AnV.

The determination of the specific extinction coefficients, K_232_ and K_268_, was carried out by preparing a 0.3% and a 1% (*m*/*v*) sample oil solution in isooctane [63]. Absorbance at 232 nm and 268 nm was measured using a UV/VIS double-beam scanning spectrophotometer (Shimadzu, Kyoto, Japan). K_232_ and K_268_ were calculated using Equation (5):

(5) $$K_{\lambda }=\frac{E_{\lambda }}{c}\times s$$

where *K_λ_* is the specific extinction coefficient at wavelength λ, *E_λ_* is the measured absorbance at wavelength λ, c is the concentration of the oil solution (g/100 mL), and *s* is the cuvette thickness (cm).

#### 3.2.5. Oxidative Stability Determination

Oxidative stability was assessed using pressure differential scanning calorimetry with a DSC Q20P thermal analyser (TA Instruments, New Castle, DE, USA). Oil sample (3.0–4.0 mg) was placed in an open aluminium pan in a cell with an empty reference pan and analysed at a constant temperature of 120 °C under a pressure of approximately 1400 kPa. The PDSC oxidation time, corresponding to the maximum rate of heat flow, was recorded and used as a measure of oxidative stability.

### 3.3. Statistical Analysis

All oil samples were analyzed in triplicate, and the results are expressed as mean ± standard deviation (SD). Statistical analysis was performed using Statistica software, version 13.3 (StatSoft, Krakow, Poland). Differences between means were evaluated using two-way analysis of variance (ANOVA), followed by Tukey’s post hoc test. Statistical significance was considered at *p* ≤ 0.05. Furthermore, multivariate analyses were employed to examine relationships between variables and to characterize the AO samples. These included PCA, HCA performed on standardized data using Ward’s method with Euclidean distance, and correlation analysis, presented as a heatmap of Pearson correlation coefficients.

## 4. Conclusions

This study demonstrated that the type of almond raw material used and the extraction method influenced the yield and quality of the obtained oils. The Folch and cold solvent extraction methods applied to blanched almonds yielded oils with the highest oleic acid and MUFA content, while commercially refined oil showed the lowest. All extracted oils had a favorable FA profile, as reflected by low indices of atherogenicity and thrombogenicity and a high h/H ratio, confirming their health-promoting potential. However, the extraction method significantly influenced the quality parameters of the obtained AOs. Those extracted by the Folch method consistently showed the highest AV, PV, *p*-AnV, TOTOX, K_232_, and K_268_ values, as well as the shortest τ_max_, indicating both their reduced oxidative stability and a more advanced state of lipid degradation. In contrast, oils obtained by the Soxhlet and cold solvent extraction methods were characterized by lower oxidation rates, with Soxhlet-extracted oils generally having the most favorable PV and K_232_ values. These studies highlight the crucial impact of the extraction method on oil stability and the need for careful selection of extraction conditions to maintain its quality.

## Figures and Tables

**Table 1:** Extraction yield (*w*/*w*, %) of almond oil obtained using different extraction methods.

Extraction Method	AO_UA	AO_BA	AO_AF	AO_APC
Soxhlet extraction	37.56 ± 1.52 ^b^	46.42 ± 2.66 ^a^	43.12 ± 2.21 ^a^	3.78 ± 0.19 ^c^
Folch extraction	37.66 ± 2.68 ^b^	42.55 ± 1.03 ^b^	40.20 ± 1.76 ^a^	7.51 ± 0.16 ^a^
Cold solvent extraction	43.43 ± 1.91 ^a^	37.02 ± 2.03 ^c^	41.42 ± 2.47 ^a^	5.72 ± 0.39 ^b^

AO_UA—oil extracted from unpeeled almonds; AO_BA—oil extracted from blanched almonds; AO_AF—oil extracted from almond flakes; AO_APC—oil extracted from almond protein concentrate. The different lower-case letters indicate significantly different values (*p* ≤ 0.05). Data are presented as mean values followed by standard deviation (±SD).

**Table 2:** Fatty acid composition (% of total fatty acids) of almond oils obtained by different extraction methods and compared with commercial refined almond oil.

	Cold Solvent Extraction				Soxhlet Extraction				Folch Extraction				Refined Oil
	AO_UA	AO_BA	AO_AF	AO_APC	AO_UA	AO_BA	AO_AF	AO_APC	AO_UA	AO_BA	AO_AF	AO_APC	AO_ref
Palmitic C16:0	7.34 ±0.02 ^b^	6.78 ±0.17 ^ef^	7.10 ±0.05 ^cd^	6.68 ±0.03 ^f^	7.23 ±0.02 ^bc^	7.12 ±0.03 ^cd^	7.09 ±0.08 ^d^	6.67 ±0.03 ^f^	7.42 ±0.03 ^a^	6.85 ±0.06 ^e^	6.91 ±0.05 ^e^	7.37 ±0.05 ^a^	5.19 ±0.01 ^f^
Palmitoleic C16:1	0.68 ±0.01 ^a^	0.51 ±0.01 ^f^	0.68 ±0.01 ^a^	0.65 ±0.01 ^cd^	0.68 ±0.01 ^a^	0.52 ±0.01 ^f^	0.64 ±0.01 ^d^	0.64 ±0.01 ^d^	0.68 ±0.01 ^a^	0.51 ±0.01 ^f^	0.66 ±0.01 ^bc^	0.60 ±0.01 ^e^	0.14 ±0.01 ^g^
Heptadecenoic C17:1	0.13 ±0.01 ^ab^	0.14 ±0.01 ^a^	0.13 ±0.01 ^ab^	0.12 ±0.01 ^cd^	0.13 ±0.01 ^ab^	0.12 ±0.01 ^cd^	0.13 ±0.01 ^ab^	0.11 ±0.01 ^d^	0.14 ±0.01 ^a^	0.12 ±0.01 ^cd^	0.13 ±0.01 ^ab^	0.11 ±0.01 ^d^	ND
Stearic C18:0	2.44 ±0.01 ^h^	2.69 ±0.01 ^g^	2.33 ±0.01 ^j^	3.39 ±0.01 ^c^	2.43 ±0.01 ^hi^	2.44 ±0.02 ^h^	2.97 ±0.03 ^e^	3.33 ±0.04 ^d^	2.41 ±0.02 ^hi^	2.81 ±0.01 ^f^	2.40 ±0.01 ^i^	3.50 ±0.01 ^b^	3.83 ±0.01 ^a^
Oleic C18:1 n-9	66.93 ±0.04 ^f^	69.79 ±0.23 ^a^	68.34 ±0.06 ^e^	69.18 ±0.05 ^c^	66.99 ±0.03 ^f^	68.66 ±0.06 ^d^	66.89 ±0.16 ^f^	69.32 ±0.02 ^c^	66.64 ±0.03 ^g^	69.59 ±0.06 ^b^	68.73 ±0.06 ^d^	68.71 ±0.03 ^d^	60.92 ±0.10 ^h^
Linoleic C18:2 n-6	22.38 ±0.04 ^c^	19.97 ±0.02 ^g^	21.33 ±0.01 ^e^	19.83 ±0.02 ^h^	22.41 ±0.01 ^c^	21.05 ±0.01 ^f^	22.14 ±0.04 ^d^	19.77 ±0.04 ^h^	22.58 ±0.01 ^b^	19.97 ±0.01 ^g^	21.08 ±0.01 ^f^	19.50 ±0.01 ^i^	27.60 ±0.07 ^a^
Arachidic C20:0	0.13 ±0.01 ^gh^	0.15 ±0.01 ^de^	0.11 ±0.01 ^i^	0.17 ±0.01 ^c^	0.14 ±0.01 ^fg^	0.12 ±0.01 ^hi^	0.15 ±0.01 ^ef^	0.16 ±0.01 ^cd^	0.15 ±0.01 ^ef^	0.16 ±0.01 ^cd^	0.11 ±0.01 ^i^	0.23 ±0.01 ^b^	0.37 ±0.01 ^a^
other	ND	ND	ND	ND	ND	ND	ND	ND	ND	ND	ND	ND	1.97 ±0.01
Σ SFA	9.90 ±0.01	9.62 ±0.18	9.54 ±0.05	10.23 ±0.03	9.79 ±0.01	9.67 ±0.06	10.21 ±0.11	10.16 ±0.01	9.97 ±0.01	9.82 ±0.05	9.41 ±0.04	11.09 ±0.04	11.06 ±0.01
Σ MUFA	67.74 ±0.04	70.43 ±0.21	69.15 ±0.05	69.95 ±0.05	67.46 ±0.02	67.66 ±0.14	69.52 ±0.05	70.06 ±0.03	67.80 ±0.02	70.21 ±0.06	69.52 ±0.05	69.42 ±0.04	61.35 ±0.08
Σ PUFA	22.38 ±0.04	19.97 ±0.02	21.33 ±0.01	19.83 ±0.02	19.77 ±0.04	22.14 ±0.04	21.08 ±0.01	19.50 ±0.01	22.58 ±0.01	19.97 ±0.01	21.08 ±0.01	19.50 ±0.01	27.60 ±0.07

AO_UA—oil extracted from unpeeled almonds; AO_BA—oil extracted from blanched almonds; AO_AF—oil extracted from almond flakes; AO_APC—oil extracted from almond protein concentrate; AO_ref—commercial, refined oil; MUFA—monounsaturated fatty acids; PUFA—polyunsaturated fatty acids; SFA—saturated fatty acids; AI—index of atherogenicity; TI—index of thrombogenicity; h/H—hypocholesterolaemic/hypercholesteraemic index. ND—not detected. Data are presented as mean values followed by standard deviation (±SD). Values in the same row with different lowercase letters (a–j) indicate a significant difference at the significance level of 0.05 (Tukey HSD).

**Table 3:** Qualitative parameters of commercial and self-extracted almond oils.

Samples	Oil Source	Acid Value (mg KOH/g)	Peroxide Value (mEq O_2_/kg)	*p*-Anisidine Value	TOTOX	Specific Extinction Coefficients	
						K_232_	K_268_
AO_UA	Cold solvent extraction	0.26 ± 0.01 ^f^	2.89 ± 0.13 ^f^	0.58 ± 0.23 ^ef^	6.36 ± 0.50 ^f^	2.00 ± 0.02 ^j^	0.26 ± 0.01 ^ef^
AO_BA		0.89 ± 0.03 ^d^	3.87 ± 0.16 ^e^	0.45 ± 0.03 ^ef^	8.19 ± 0.36 ^e^	2.51 ± 0.06 ^h^	0.13 ± 0.01 ^g^
AO_AF		1.49 ± 0.03 ^c^	2.05 ± 0.06 ^g^	0.30 ± 0.01 ^f^	4.40 ± 0.11 ^g^	2.67 ± 0.11 ^gh^	0.10 ± 0.01 ^g^
AO_APC		0.24 ± 0.01 ^f^	3.78 ± 0.15 ^e^	0.75 ± 0.10 ^e^	8.31 ± 0.39 ^e^	3.42 ± 0.07 ^c^	0.37 ± 0.01 ^cd^
AO_UA	Soxhlet extraction	0.25 ± 0.02 ^f^	1.77 ± 0.21 ^g^	1.22 ± 0.20 ^d^	4.77 ± 0.62 ^g^	1.89 ± 0.06 ^j^	0.27 ± 0.01 ^ef^
AO_BA		0.90 ± 0.01 ^d^	1.99 ± 0.01 ^g^	0.51 ± 0.12 ^ef^	4.48 ± 0.14 ^g^	2.19 ± 0.01 ^i^	0.12 ± 0.01 ^g^
AO_AF		1.37 ± 0.04 ^c^	1.87 ± 0.26 ^g^	0.62 ± 0.01 ^ef^	4.35 ± 0.51 ^g^	2.64 ± 0.04 ^gh^	0.12 ± 0.01 ^g^
AO_APC		0.40 ± 0.02 ^ef^	2.25 ± 0.06 ^g^	1.54 ± 0.21 ^d^	6.04 ± 0.09 ^f^	3.18 ± 0.04 ^d^	0.41 ± 0.01 ^c^
AO_UA	Folch extraction	0.74 ± 0.04 ^de^	10.19 ± 0.20 ^b^	1.93 ± 0.13 ^c^	22.31 ± 0.53 ^b^	2.87 ± 0.01 ^ef^	0.40 ± 0.01 ^c^
AO_BA		1.50 ± 0.11 ^c^	5.04 ± 0.07 ^d^	3.84 ± 0.15 ^a^	13.92 ± 0.30 ^d^	2.75 ± 0.15 ^fg^	0.23 ± 0.01 ^f^
AO_AF		2.04 ± 0.10 ^b^	5.74 ± 0.12 ^c^	3.99 ± 0.03 ^a^	15.47 ± 0.27 ^c^	2.99 ± 0.06 ^e^	0.31 ± 0.03 ^de^
AO_APC		16.77 ± 0.69 ^a^	13.07 ± 0.13 ^a^	3.98 ± 0.11 ^a^	30.11 ± 0.15 ^a^	4.97 ± 0.19 ^b^	0.86 ± 0.02 ^b^
AO_ref	Refined Oil	0.24 ± 0.01 ^f^	9.65 ± 0.67 ^b^	3.24 ± 0.39 ^b^	22.53 ± 0.94 ^b^	5.83 ± 0.05 ^a^	1.24 ± 0.12 ^a^

AO_UA—oil extracted from unpeeled almonds; AO_BA—oil extracted from blanched almonds; AO_AF—oil extracted from almond flakes; AO_APC—oil extracted from almond protein concentrate; AO_ref—commercial, refined oil. The data are the means of three independent experiments ± standard deviations (n = 3). Values in the same column with different lowercase letters (a–j) indicate a significant difference at the significance level of 0.05 (Tukey HSD).

## Data Availability

Data are contained within the article.

## Supplementary Materials

The following supporting information can be downloaded at: https://www.mdpi.com/article/10.3390/molecules30173519/s1, Table S1. Health indices of almond oils obtained by different extraction methods and compared 1 with commercial refined almond oil.

## Abbreviations

The following abbreviations are used in this manuscript:

AI	Index of atherogenicity
AO	Almond oil
AO_AF	Oil extracted from almond flakes
AO_APC	Oil extracted from almond protein concentrate
AO_BA	Oil extracted from blanched almonds
AO_ref	Commercial, refined almond oil
AO_UA	Oil extracted from unpeeled almonds
AOCS	American Oil Chemists’ Society
APC	Almond protein concentrate
AV	Acid value
CSE	Cold solvent extraction
FA	Fatty acid
FE	Folch extraction
h/H	Hypocholesterolaemic/hypercholesteraemic index
HCA	Hierarchical Cluster Analysis
K_232_	Specific extinction coefficient at 232 nm
K_268_	Specific extinction coefficient at 268 nm
LDL	Low-density lipoprotein
MUFA	Monounsaturated fatty acids
ND	Not detected
*p*-AnV	*p*-Anisidine value
PC1	First principal component
PC2	Second principal component
PCA	Principal Component Analysis
PDSC	Pressure Differential Scanning Calorimetry
PUFA	Polyunsaturated fatty acids
PV	Peroxide value
SD	Standard deviation
SE	Soxhlet extraction
SFA	Saturated fatty acids
TI	Index of thrombogenicity
TOTOX	Total oxidation index
τ_max_	PDSC oxidation time

## References

[1] Gharehyakheh S. (2022). Optimization of Bitter Almond Oil (BAO) Extraction Conditions Using Natural Enzymes and Ultrasound Waves. Iran. J. Chem. Eng..

[2] Berkkan A., Türk B.N.D., Pekacar S., Ulutaş O.K., Orhan D.D. (2022). Evaluation of Marketed Almond Oils [*Prunus dulcis* (Mill.) D.A. Webb] in Terms of European Pharmacopoeia Criteria. Turk. J. Pharm. Sci..

[3] Tian L., You X., Zhang S., Zhu Z., Yi J., Jin G. (2024). Enhancing Functional Properties and Protein Structure of Almond Protein Isolate Using High-Power Ultrasound Treatment. Molecules.

[4] Yildirim A.N., Akinci-Yildirim F., San B., Sesli Y. (2016). Total Oil Content and Fatty Acid Profile of Some Almond (*Amygdalus communis* L.) Cultivars. Pol. J. Food Nutr. Sci..

[5] El Bernoussi S., Boujemaa I., Harhar H., Belmaghraoui W., Matthäus B., Tabyaoui M. (2020). Evaluation of Oxidative Stability of Sweet and Bitter Almond Oils under Accelerated Storage Conditions. J. Stored Prod. Res..

[6] Siddiqua A., Hussain S., Syed S.K. (2021). Phytochemistry, Nutritional and Medicinal Importance of Almond. Postep. Biol. Komorki.

[7] Oliveira I., Meyer A.S., Afonso S., Aires A., Goufo P., Trindade H., Gonçalves B. (2019). Phenolic and Fatty Acid Profiles, α-Tocopherol and Sucrose Contents, and Antioxidant Capacities of Understudied Portuguese Almond Cultivars. J. Food Biochem..

[8] Ouzir M., Bernoussi S.E., Tabyaoui M., Taghzouti K. (2021). Almond Oil: A Comprehensive Review of Chemical Composition, Extraction Methods, Preservation Conditions, Potential Health Benefits, and Safety. Compr. Rev. Food Sci. Food Saf..

[9] Martín-Tornero E., Simón-García D., Álvarez-Ortí M., Pardo J.E., Durán-Merás I., Martín-Vertedor D. (2024). Non-Destructive Fluorescence Spectroscopy for Quality Evaluation of Almond Oils Extracted from Roasted Kernel. Talanta Open.

[10] Dias F.F.G., Teixeira B.F., Taha A.Y., Bell J.M.L.N.d.M. (2025). Integrated Impact of Environmentally Friendly Extraction and Recovery Methods on Almond Oil Quality: Insights from a Lipidomic Perspective. J. Am. Oil Chem. Soc..

[11] Qi Z., Xiao J., Ye L., Chuyun W., Chang Z., Shugang L., Fenghong H. (2019). The Effect of the Subcritical Fluid Extraction on the Quality of Almond Oils: Compared to Conventional Mechanical Pressing Method. Food Sci. Nutr..

[12] Melhaoui R., Kodad S., Houmy N., Belhaj K., Mansouri F., Abid M., Addi M., Mihamou A., Sindic M., Serghini-Caid H. (2021). Characterization of Sweet Almond Oil Content of Four European Cultivars (*Ferragnes*, *Ferraduel*, *Fournat*, and *Marcona*) Recently Introduced in Morocco. Scientifica.

[13] Ruchi V., Nayanjeet C., Kalra P., Nair N.S., Prabhakar B. (2024). Effects of Almond Consumption Compared with the Consumption of Traditional Isocaloric Cereal/Pulse-Based Snacks on Glycaemic Control and Gut Health in Adults with Pre-Diabetes in Rural India: Protocol for a 16-Week, Parallel-Arm, Cluster Randomised Controlled Trial. BMJ Open.

[14] Özcan M.M., Al Juhaimi F., Ghafoor K., Babiker E.E., Özcan M.M. (2020). Characterization of Physico-Chemical and Bioactive Properties of Oils of some Important Almond Cultivars by Cold Press and Soxhlet Extraction. J. Food Sci. Technol..

[15] Sayah O., Taibi S., Bouakline H., El Yousfi R., Tayebi A., Ziani I., Tahani A., El Bachiri A. (2025). Comparison of Sweet and Bitter Almond Oil Quality: Impact of Kernel Skin and Green Supercritical CO_2_ Extraction Method. Food Chem..

[16] Oliveira I., Meyer A.S., Afonso S., Sequeira A., Vilela A., Goufo P., Trindade H., Gonçalves B. (2020). Effects of Different Processing Treatments on Almond (*Prunus dulcis*) Bioactive Compounds, Antioxidant Activities, Fatty Acids, and Sensorial Characteristics. Plants.

[17] Nde D.B., Foncha A.C. (2020). Optimization Methods for the Extraction of Vegetable Oils: A Review. Processes.

[18] Danlami J.M., Arsad A., Zaini M.A.A., Sulaiman H. (2014). A Comparative Study of Various Oil Extraction Techniques from Plants. Rev. Chem. Eng..

[19] Kozłowska M., Gruczyńska E., Ścibisz I., Rudzińska M. (2016). Fatty Acids and Sterols Composition, and Antioxidant Activity of Oils Extracted from Plant Seeds. Food Chem..

[20] Krzyczkowska J., Kozłowska M. (2017). Effect of Oils Extracted from Plant Seeds on the Growth and Lipolytic Activity of *Yarrowia lipolytica* Yeast. J. Am. Oil Chem. Soc..

[21] Gambert A., Niţu S., Tămaș A., Fanani M., Dupré J., Delepine C., Chaveriat L., Martin P., Rusnac L. (2024). Influence of the Extraction Process on the Characteristics of Romanian Mountain Walnut Oil. Am. J. Plant Sci..

[22] Miraliakbari H., Shahidi F. (2008). Lipid Class Compositions, Tocopherols and Sterols of Tree Nut Oils Extracted with Different Solvents. J. Food Lipids.

[23] Siol M., Witkowska B., Mańko-Jurkowska D., Makouie S., Bryś J. (2025). Comprehensive Evaluation of the Nutritional Quality of Stored Watermelon Seed Oils. Appl. Sci..

[24] Chen J., Liu H. (2020). Nutritional Indices for Assessing Fatty Acids: A Mini-Review. Int. J. Mol. Sci..

[25] Stolp L.J., Kodali D.R., Flider F.J. (2022). Chapter 2—Naturally occurring high-oleic oils: Avocado, macadamia, and olive oils. High Oleic Oils.

[26] Gülsoy E., Kaya E.D., Türkhan A., Bulut M., Koyuncu M., Güler E., Sayın F., Muradoğlu F. (2023). The Effect of Altitude on Phenolic, Antioxidant and Fatty Acid Compositions of Some Turkish Hazelnut (*Coryllus avellana* L.) Cultivars. Molecules.

[27] Poli A., Agostoni C., Visioli F. (2023). Dietary Fatty Acids and Inflammation: Focus on the n-6 Series. Int. J. Mol. Sci..

[28] Maestri D., Cittadini M.C., Bodoira R., Martínez M. (2020). Tree Nut Oils: Chemical Profiles, Extraction, Stability, and Quality Concerns. Eur. J. Lipid Sci. Technol..

[29] Rabadán A., Pardo J.E., Gómez R., Álvarez-Ortí M. (2018). Effect of Almond Roasting, Light Exposure and Addition of Different Garlic Cultivars on Almond Oil Stability. Eur. Food Res. Technol..

[30] Tian M., Bai Y., Tian H., Zhao X. (2023). The Chemical Composition and Health-Promoting Benefits of Vegetable Oils—A Review. Molecules.

[31] Tilami S.K., Kouřimská L. (2022). Assessment of the Nutritional Quality of Plant Lipids Using Atherogenicity and Thrombogenicity Indices. Nutrients.

[32] Ying Q., Wojciechowska P., Siger A., Kaczmarek A., Rudzińska M. (2018). Phytochemical Content, Oxidative Stability, and Nutritional Properties of Unconventional Cold-pressed Edible Oils. J. Food Nutr. Res..

[33] Wirkowska-Wojdyła M., Ostrowska-Ligęza E., Górska A., Brzezińska R., Piasecka I. (2024). Assessment of the Nutritional Potential and Resistance to Oxidation of Sea Buckthorn and Rosehip Oils. Appl. Sci..

[34] Ratusz K., Symoniuk E., Wroniak M., Rudzińska M. (2018). Bioactive Compounds, Nutritional Quality and Oxidative Stability of Cold-Pressed Camelina (*Camelina sativa* L.) Oils. Appl. Sci..

[35] (2009). Codex Alimentarius 2009. Codex Standard for Named Vegetable Oils.

[36] Gharby S., Hajib A., Ibourki M., Sakar E.H., Nounah I., Moudden H.E., Elibrahimi M., Harhar H. (2021). Induced Changes in Olive Oil Subjected to Various Chemical Refining Steps: A Comparative Study of Quality Indices, Fatty Acids, Bioactive Minor Components, and Oxidation Stability Kinetic Parameters. Chem. Data Collect..

[37] Gharby S. (2022). Refining Vegetable Oils: Chemical and Physical Refining. Sci. World J..

[38] Atamyradova N., Özkılıç S.Y., Arslan D. (2024). Blanching of Olive Fruits Before Storage at Different Conditions: Effects on Oil Yield, Lipase Activity and Oxidation. J. Agric. Food Res..

[39] (2017). Vegetable and Animal Oils and Fats. Determination of Peroxide Number (Reference Method)..

[40] Rezvankhah A., Emam-Djomeh Z., Safari M., Askari G., Salami M. (2019). Microwave-Assisted Extraction of Hempseed Oil: Studying and Comparing of Fatty Acid Composition, Antioxidant Activity, Physiochemical and Thermal Properties with Soxhlet Extraction. J. Food Sci. Technol..

[41] Mortensen G., Sørensen J., Stapelfeldt H. (2002). Comparison of Peroxide Value Methods used for Semihard Cheeses. J. Agric. Food Chem..

[42] Esfahani S.T., Zamindar N., Esmaeili Y., Sharifian S. (2024). Effect of Initial Quality of Oil and Thermal Processing on Oxidation Indexes in Canned Tuna. Appl. Food Res..

[43] Čolić S., Zec G., Natić M., Fotirić-Akšić M., Ramadan M. (2019). Almond (*Prunus dulcis*) oil. Fruit Oils: Chemistry and Functionality.

[44] Sidhu A.R., Naz S., Mahesar S.A., Kandhro A.A., Khaskheli A.R., Ali Z., Memon H.D., Shoaib H., Mahesar H.R. (2023). Effect of Storage at Elevated Temperature on the Quality and Stability of Different Almond Oils: A Comprehensive Study. Food Mater. Res..

[45] Petkova Z., Antova G. (2019). A Comparative Study on Quality Parameters of Pumpkin, Melon and Sunflower Oils During Thermal Treatment. OCL.

[46] Sánchez-Bel P., Martínez-Madrid M.C., Egea I., Romojaro F. (2005). Quality and Sensory Evaluation of Almond (*Prunus amygdalus*) Stored after Electron Beam Processing. J. Agric. Food Chem..

[47] Tan C.P., Man Y.B.C., Selamat J., Yusoff M.S.A. (2002). Comparative Studies of Oxidative Stability of Edible Oils by Differential Scanning Calorimetry and Oxidative Stability Index Methods. Food Chem..

[48] Buranasompob A., Tang J., Powers J.R., Reyes J., Clark S., Swanson B.G. (2007). Lipoxygenase Activity in Walnuts and Almonds. LWT Food Sci. Technol..

[49] Mwaurah P.W., Kumar S., Kumar N., Attkan A.K., Panghal A., Singh V.K., Garg M.K. (2020). Novel Oil Extraction Technologies: Process Conditions, Quality Parameters, and Optimization. Compr. Rev. Food Sci. Food Saf..

[50] Magangana T.P., Makunga N.P., la Grange C., Stander M.A., Fawole O.A., Opara U.L. (2021). Blanching Pre-Treatment Promotes High Yields, Bioactive Compounds, Antioxidants, Enzyme Inactivation and Antibacterial Activity of ‘Wonderful’ Pomegranate Peel Extracts at Three Different Harvest Maturities. Antioxidants.

[51] Salcedo C.L., de Mishima B.A.L., Nazareno M.A. (2010). Walnuts and Almonds as Model Systems of Foods Constituted by Oxidisable, Pro-Oxidant and Antioxidant Factors. Food Res. Int..

[52] Symoniuk E., Wroniak M., Napiórkowska K., Brzezińska R., Ratusz K. (2022). Oxidative Stability and Antioxidant Activity of Selected Cold-Pressed Oils and Oils Mixtures. Foods.

[53] Ratusz K., Kowalski B., Bekas W., Wirkowska M. (2005). Monitorowanie Autooksydacji Oleju Rzepakowego i Słonecznikowego. Rośliny Oleiste Oilseed Crops.

[54] Orsavova J., Misurcova L., Ambrozova J.V., Vicha R., Mlcek J. (2015). Fatty Acids Composition of Vegetable Oils and Its Contribution to Dietary Energy Intake and Dependence of Cardiovascular Mortality on Dietary Intake of Fatty Acids. Int. J. Mol. Sci..

[55] Folch J., Lees M., Stanley G.H.S. (1957). A Simple Method for the Isolation and Purification of Total Lipides from Animal Tissues. J. Biol. Chem..

[56] Boselli E., Velazco V., Caboni M.F., Lercker G. (2001). Pressurized Liquid Extraction of Lipids for the Determination of Oxysterols in Egg-Containing Food. J. Chromatogr. A.

[57] (2001). Vegetable and Animal Oils and Fats. Preparation of Fatty Acid Methyl Esters..

[58] Ulbricht T.L.V., Southgate D.A.T. (1991). Coronary Heart Disease: Seven Dietary Factors. Lancet.

[59] Santos-Silva J., Bessa R.J.B., Santos-Silva F. (2022). Effect of Genotype, Feeding System and Slaughter Weight on the Quality of Light Lambs: II. Fatty Acid Composition of Meat. Livest. Prod. Sci..

[60] (2009). Acid Value Official Methods and Recommended Practices of the AOCS.

[61] (2009). Peroxide Value Acetic Acid-Isooctane Method Official Methods and Recommended Practices of the AOCS.

[62] AOCS Official Method Cd 18-90 (2011). *p*-Anisidine Value. Official Methods and Recommended Practices of the AOCS.

[63] (2011). Animal and Vegetable Fats and Oils-Determination of Ultraviolet Absorbance Expressed as Specific UV Extinction.

