# Supplementary material for: Influence of Extraction Techniques on Almond Oil Quality: A Comparative Study of Solvent-Extracted and Commercial Products
Source: Molecules. 2025 Aug 28;30(17):3519. doi: 10.3390/molecules30173519 (PMC12429927; doi:10.3390/molecules30173519)
Supplement: Supplementary file 1 [file molecules-30-03519-s001.zip › molecules-3805540-supplementary.pdf]

Table S1. Health indices of almond oils obtained by different extraction methods and compared with commercial refined almond oil.

Samples	Extraction method	AI	TI	h/H
AO_UA	Cold solvent extraction	0.08	0.22	12.17
AO_BA		0.08	0.21	13.24
AO_AF		0.08	0.21	12.64
AO_APC		0.07	0.22	13.32
AO_UA	Soxhlet extraction	0.08	0.21	12.37
AO_BA		0.08	0.21	12.60
AO_AF		0.08	0.22	12.56
AO_APC		0.07	0.22	13.36
AO_UA	Folch extraction	0.08	0.22	12.02
AO_BA		0.08	0.21	13.08
AO_AF		0.08	0.21	13.01
AO_APC		0.08	0.24	11.98
AO_ref	Refined Oil	0.08	0.21	12.64

AO_UA – oil extracted from unpeeled almonds; AO_BA – oil extracted from blanched almonds; AO_AF – oil extracted from almond flakes; AO_APC – oil extracted from almond protein concentrate; AO_ref – commercial, refined oil; AI – index of atherogenicity; TI – index of thrombogenicity; h/H – hypocholesterolaemic/hypercholesteraemic index.
